# The effects of TMS over the anterior intraparietal area on anticipatory fingertip force scaling and the size-weight illusion

**DOI:** 10.1152/jn.00265.2021

**Published:** 2022-03-16

**Authors:** Vonne van Polanen, Gavin Buckingham, Marco Davare

**Affiliations:** ^1^Movement Control and Neuroplasticity Research Group, Department of Movement Sciences, Biomedical Sciences group, KU Leuven, Leuven, Belgium; ^2^Leuven Brain Institute, KU Leuven, Leuven, Belgium; ^3^Department of Sport and Health Sciences, University of Exeter, Exeter, United Kingdom; ^4^Faculty of Life Sciences and Medicine, King’s College London, London, United Kingdom

**Keywords:** force scaling, grasping, parietal cortex, size-weight illusion, TMS

## Abstract

When lifting an object skillfully, fingertip forces need to be carefully scaled to the object’s weight, which can be inferred from its apparent size and material. This anticipatory force scaling ensures smooth and efficient lifting movements. However, even with accurate motor plans, weight perception can still be biased. In the size-weight illusion, objects of different size but equal weight are perceived to differ in heaviness, with the small object perceived to be heavier than the large object. The neural underpinnings of anticipatory force scaling to object size and the size-weight illusion are largely unknown. In this study, we tested the role of anterior intraparietal cortex (aIPS) in predictive force scaling and the size-weight illusion, by applying continuous theta burst stimulation (cTBS) prior to participants lifting objects of different sizes. Participants received cTBS over aIPS, the primary motor cortex (control area), or Sham stimulation. We found no evidence that aIPS stimulation affected the size-weight illusion. Effects were, however, found on anticipatory force scaling, where grip force was less tuned to object size during initial lifts. These findings suggest that aIPS is not involved in the perception of object weight but plays a transient role in the sensorimotor predictions related to object size.

**NEW & NOTEWORTHY** Skilled object manipulation requires forming anticipatory motor plans according to the object’s properties. Here, we demonstrate the role of anterior intraparietal sulcus (aIPS) in anticipatory grip force scaling to object size, particularly during initial lifting experience. Interestingly, this role was not maintained after continued practice and was not related to perceptual judgments measured with the size-weight illusion.

## Graphical abstract

**Figure:**
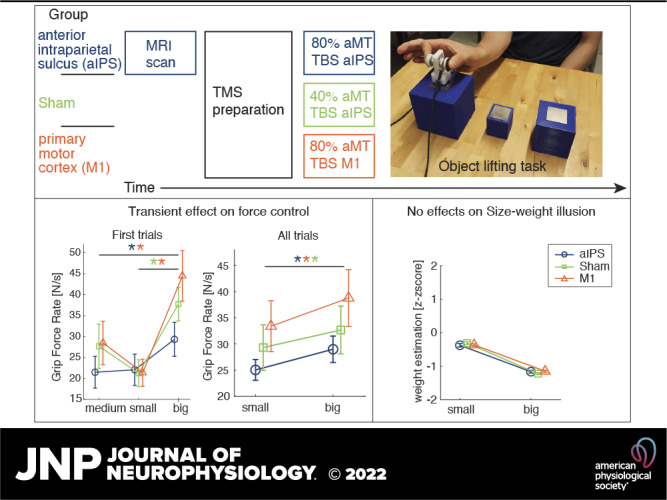

## INTRODUCTION

A key feature of skilled object manipulation in humans is the accurate planning of hand and fingertip forces. This motor plan relies on predictions of object weight from object characteristics, such as size and material. This anticipatory prediction allows for the generation of fingertip forces scaled to object weight and ensure smooth lifting movements. When the prediction is incorrect, forces can be quickly adjusted (1).

The action of lifting an object can also provide more information about the object’s properties, such as its weight. However, weight perception is not always veridical. For instance, in the size-weight illusion (SWI) (2), a smaller object is judged to be heavier than an equally weighted large object (for reviews, see Refs. 3–5). To induce the illusion, differences in object size can be perceived visually, haptically (6, 7), or even illusory (8) and can be independent of object volume (9).

Although initially both force scaling and weight perception are influenced by object size, after a few lifts, force scaling rapidly adapts to the actual weight of the objects, whereas the SWI remains constant (10–12). This might be due to sensorimotor memory for object weight, where recent experience with object lifting is used for force scaling of the next lifts (1). It has therefore been suggested that force scaling and weight perception are underpinned by different sources of information (10).

The neural networks for anticipatory force scaling to size and the SWI are largely unknown. Previous research showed that various brain areas are involved in force scaling, when object weight was different than predicted (13, 14). Functional magnetic resonance imaging (fMRI) studies showed that the posterior parietal cortex is active in force coordination, which plays a crucial role in anticipatory force scaling (15). Using transcranial magnetic stimulation (TMS), the causal role of an area in controlling a specific movement and/or perceptual parameter can be inferred. Previous TMS research identified a role of the anterior intraparietal sulcus (aIPS) in anticipatory force scaling based on sensorimotor memories, when lifting similar looking objects (16, 17). However, anticipatory force scaling can also be based on a different source of information, such as visual feedback about an object’s size; and it is unclear whether aIPS also contributes to processing an object’s visual features for controlling fingertip forces. aIPS is a good candidate, since nonhuman primate (18) and human brain imaging (19, 20) studies have shown aIPS activations related to object size, especially in the context of grasping (21) and relevant grasp dimensions (22). Furthermore, it is known that aIPS is involved in controlling grasp components in reaching with perturbations to object size (23, 24). Therefore, it seems that aIPS is not only involved in the control of force scaling, but also sensitive to object size. Since object size is often indicative of object weight, this suggests that aIPS could also be important in anticipatory force scaling to object size.

Regarding the SWI, few neuroimaging studies in healthy subjects have been performed. One fMRI study showed that the ventral premotor cortex showed greater levels of adaptation in trials that elicited the SWI compared with when lifting objects of the same size and weight, whereas aIPS mainly responded to size (19). On the other hand, clinical evidence suggests that lesions in the parietal cortex can reduce the SWI (25), although the same research group found more mixed results in another study (26).

To summarize, there is considerable evidence that suggests aIPS could play a role in anticipatory scaling to object size, but its potential contribution to the SWI is less clear. The present study aims to investigate the role of aIPS in these sensorimotor processes. Specifically, we wanted to investigate the role of aIPS in the SWI and anticipatory force scaling in response to visual information, i.e., object size. Participants lifted objects of different size and weight over multiple trials and reported their felt heaviness on each trial, while grip and load forces were measured. TMS was used to disrupt aIPS and determine its causal role in motor and perceptual processes. As control conditions, we used TMS over the primary motor cortex (M1) and a Sham stimulation over aIPS. We expected that stimulation to M1 and aIPS would affect force scaling according to object weight, with anticipatory force scaling to object size specifically altered by stimulation to aIPS. In turn, this affected force scaling to size could also reduce the mismatch between perceived and expected weight, hence leading to a reduced size-weight illusion.

## MATERIALS AND METHODS

### Participants

Forty-seven participants (27 females, 22 ± 3.3 yr old) took part in the study. They were all right-handed [Edinburgh handedness inventory, mean laterality quotient: 0.86 ± 0.19 (27)] and were screened for potential TMS risks (28). They provided written informed consent before participation. Participants were divided into three groups, one for each TMS stimulation site (aIPS: *n* = 16, M1: *n* = 16, or Sham: *n* = 15). Two participants were excluded from force analysis (one from the aIPS group and one from the M1 group), because of technical errors in the force data recording. The study was approved by the medical ethical committee of KU Leuven.

The participants were divided into three groups. Since we were interested in anticipatory force scaling to object size, which might only be visible in the very first trials when participants encounter the objects for the first time, we could only measure a participant in a single TMS session. Furthermore, the separate participant groups would ensure that they were blinded to conditions, that is, they would not be able to distinguish the Sham condition from the experimental condition (see *TMS Procedure* section).

### TMS Procedure

TMS was applied before the behavioral task using a 70-mm figure-of-eight TMS DuoMag 70BF coil connected to a DuoMag XT100 system (Deymed Diagnostic) or a Magstim rapid stimulator (Mk1, Magstim company) with a D-70 α TMS coil. We used a Brainsight system (Rogue Research, Canada) for neuronavigation and recording of electromyography (EMG). EMG was recorded in the right first dorsal interosseus (FDI) using a belly tendon montage. A ground electrode was placed on the processus styloideus ulnae. To determine the stimulation intensity, we determined the rest (rMT) and active (aMT) motor threshold of FDI when stimulating the motor hotspot, which was defined as the position on M1 that gave the largest motor-evoked potential (MEP) in response to TMS. Here, rMT was the stimulation intensity that gave MEPs of at least 50 µV in 5/10 stimulations (28, 29) while the hand was at rest and aMT the intensity that gave MEPs in 5/10 trials visibly larger than background EMG during contraction of FDI at submaximal levels. The average rMT and aMT were 55% (33%–72%) and 46% (25%–63%) stimulator output, respectively. The rMT was used to measure MEPs and the aMT to perform continuous theta burst stimulation (cTBS).

Before performing the behavioral task, the three groups of participants received cTBS to aIPS, M1, or were delivered Sham stimulation. cTBS induces a “virtual lesion” in the stimulated brain area, affecting brain activity for a longer period of time (∼1 h) (30). This stimulation protocol was chosen to induce a virtual lesion over a large time period, because it is unclear at what time point during the lifting movements aIPS would be involved in force scaling and weight perception processes. Since it is known that force scaling based on sensorimotor memory of object weight is represented in M1 (31), we chose this region as a control area to test whether TMS effects of anticipatory force scaling were not due to unspecific TMS effects or general alterations in force control. Specifically, we expected effects of M1 stimulation on force scaling based on previous object weight (i.e., sensorimotor memory), but not based on current object size (i.e., anticipatory force scaling to size). In our Sham condition, a reduced intensity of cTBS was applied on aIPS. Earlier studies showed that a reduced TMS intensity showed different or no effects compared with high intensity (32–35). Since a between-subjects design was used, participants did not know whether they received cTBS at a high (i.e., experimental condition) or low intensity (i.e., Sham condition).

**Figure 1.**
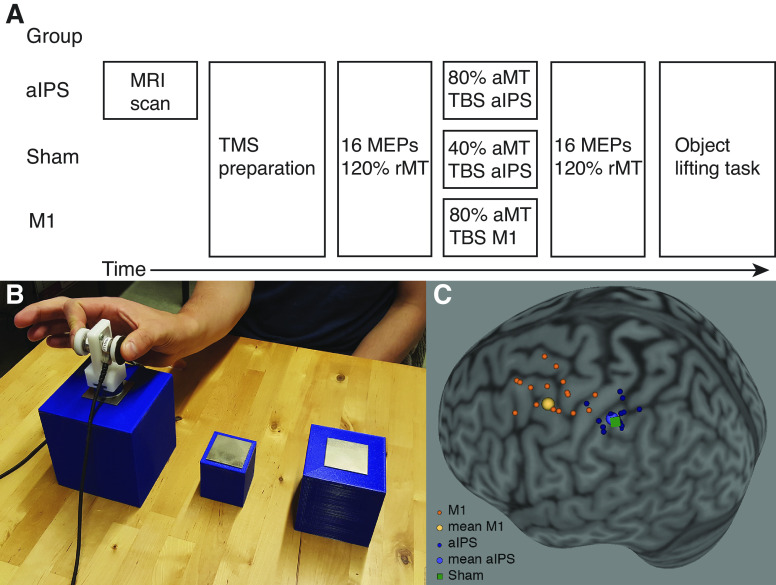
*A*: timeline of experiment. Participants were divided into the anterior intraparietal sulcus (aIPS), Sham, or M1 group. The aIPS group received an magnetic resonance imaging (MRI) scan to anatomically determine the stimulation target. Participants received continuous theta burst stimulation (cTBS) at 40% or 80% of the active motor threshold (aMT). Before and after cTBS, 16 motor-evoked potentials (MEPs) were obtained at 120% rest motor threshold (rMT). After this, participants performed a behavioral task, consisting of lifting objects of different sizes and weights. *B*: experimental setup with large, small, and medium sized objects. The medium object was only used in practice trials. For the small and large objects, there was a light and heavy set. A grip manipulandum with force sensors could be quickly attached to the objects using magnets. *C*: individual and average stimulation targets, projected on the cortex of the Colin27 MNI reference brain. Note that M1 targets and Sham targets were recorded on a model brain from Brainsight Software. TBS, theta burst stimulation.

Participants received cTBS following standard procedures (600 pulses, 50 Hz triplets at 5 Hz for a total duration of 40 ms) (30). The aIPS and M1 group received cTBS at 80% of the aMT over aIPS or M1, respectively. The Sham group received cTBS at a low intensity, namely 40% of aMT over aIPS. Stimulation locations were monitored using Brainsight (Rogue Research) software. In the aIPS group, aIPS was defined anatomically on a structural magnetic resonance imaging (MRI) scan, at the intersection of the posterior sulcus and the intraparietal sulcus. Brain images were obtained with a 3-T scanner (Achieva dstream, Phillips Medical Systems) as high-resolution three-dimensional (3-D) T1-weighted images (TR = 9.7 ms, TE = 4.6 ms, field of view = 256 × 256 mm^2^, 192 slices, voxel size = 0.98 × 0.98 × 1.2 mm^3^). The mean MNI coordinates of aIPS in the aIPS group were (−45 ± 3, −39 ± 6, 48 ± 5), which is close to previously reported values (21, 22, 36). For the Sham and M1 groups, no MR image was obtained, but targets were determined on a model brain from the Brainsight software. The Sham target was defined on a model brain with aIPS MNI coordinates from literature (−43, −39, 46) (36). Finally, for M1, the motor hotspot also used for recording MEPs was used (mean MNI coordinates: −64 ± 14, 2 ± 16, 77 ± 7, as recorded on a model brain). Individual and average stimulation sites are shown in Fig. 1*C*, where all targets are shown projected on the cortex. Note that the coordinates from M1 were recorded from the skin, whereas the aIPS targets were determined on the cortex. In addition, the M1 targets were recorded on a model brain, therefore, the individual targets are much more variable. In all three groups, the coil was positioned with the handle pointing backward, roughly 45° from the midline, with a posterior-anterior current direction.

To measure the effects of cTBS on corticospinal excitability, we collected MEPs. Before cTBS, 16 MEPs were collected by stimulating over M1 at an intensity of 120% rMT (pre-MEPs). Next, cTBS was performed after which a 5-min rest period was induced, where the hand did not move. This rest period was introduced because clear effects of cTBS appear after 5 min (30). After this rest period, another 16 MEPs were measured with M1 stimulation at 120% rMT (post-MEPs). Following the TMS procedure, participants performed the behavioral task within 45 min after the cTBS. Therefore, the task was completed within the effective period of cTBS as reported in literature (30). The timeline of the experiment is illustrated in Fig. 1*A*.

### Grasp and Lift Task

Four different 3-D-printed cubes were used in the experiment, two large and two small ones. The large cubes measured 10 cm × 10 cm × 10 cm and the small ones 5 cm × 5 cm × 5 cm. The objects were filled with lead shot to create two different weights, 190 and 400 g. Therefore, four objects were used: small-light, small-heavy, large-light, and large-heavy. A fifth object of a medium size (7.5 cm × 7.5 cm × 7.5 cm, weight 190 g) was used for practice trials. A pair of force sensors (Nano17, ATI Industrial Automation) was fastened to a 3-D-printed manipulandum (Fig. 1*B*). The manipulandum had three magnets to quickly attach it to metal squares of 5 cm × 5 cm that were glued on top of the objects. The objects were placed behind a screen (Magic Glass) that could switch between a transparent and an opaque state.

Participants were instructed to grasp and lift the cube by placing the thumb and index finger on the force sensors. They could lift the cube as soon as the screen turned transparent and the object was visible. Then, they should lift it to a height of ∼5 cm and hold it in the air until the screen turned opaque again (±3 s), upon which they replaced the object on the table. To measure the perceptual estimates of object weight, we used the method of magnitude estimation (37). After participants replaced the object, they were asked to give a number best representing the weight of the object on a self-chosen scale without a predetermined upper or lower limit. These self-chosen scales were normalized in the data analysis by converting the answers to *z* scores.

Each of the four cubes was presented 20 times in a pseudo-randomized order. Of these 20 trials, each object was followed by a small or large object (of any weight) 10 times, respectively. This was done to make alterations in size from trial to trial approximately equal across the experiment. Since the first trial was not preceded by any object, one randomly chosen object was added to the trials. This gave a total of 81 trials (4 objects × 20 trials +1). To be able to compare the effect of object appearance (i.e., size) before any experience with the objects, the first two lifts were always performed with the small-light and large-light object. Before the start of the experiment, participants performed 10 practice trials with the medium cube.

### Data Analysis

#### MEPs.

MEPs were calculated using Brainsight software as the peak-to-peak amplitude of the EMG response. From the 16 MEPs, the first was removed to exclude surprise effects and the remaining 15 were averaged for the pre- and postsession, respectively. Three MEPs (all in the post-MEP session) were removed because the coil was off-target (>3 mm). One participant was excluded from this analysis (M1 group), due to large TMS artifacts in the post-MEP session.

#### Force and perceptual analysis.

Trials were removed from the perception and force analysis when objects were lifted twice, the object was dropped, or when it was touched before the screen turned transparent (15 trials). Since some of these trials were the first or second trials, three participants were excluded from the first-trial analysis for both the perceptual and force parameters. Two participants were completely excluded (one from aIPS group and one from M1 group) from the force analysis due to technical errors in force data collection. The number of participants used in the data analysis is indicated in all figures.

The weight judgments from participants were converted to *z* scores to normalize the values, which were confirmed by a Shapiro–Wilk test. To smoothen the signal, forces were filtered with a second-order bidirectional lowpass Butterworth filter with a cut-off frequency of 15 Hz. Grip forces (GF) were defined as the mean of the forces perpendicular to the sensor, and load forces (LF) were the sum of the vertical forces. Force rates were the first-order differentiated forces with respect to time, GFR, and LFR, respectively. Parameters of interest were the first peak of GFR (GFR1st), the first peak of LFR (LFR1st), and the loading phase duration (LPD). The first peak of the force rates were the first peaks after grip force onset (GF > 0.1 N and further increasing to 0.8 N). To exclude peaks due to noise and positioning the fingers on the sensors, only peaks that were at least 70% of the maximum force rates were included, in accordance with the procedure in Ref. 38. The loading phase duration was the time between LF onset (LF > 0.1 N and further increasing to 0.8 N) and lift-off (LF > object weight).

#### Statistics.

Statistics were performed with SPSS version 27 (IBM). Paired-samples *t* tests were used to determine differences between pre- and post-MEPs for each group. Force parameters and perceptual estimates were analyzed in three ways. First, we looked at the behavioral effects of size and weight averaged across all lifts for each object. The parameters (GFR1st, LFR1st, LPD, and perceptual estimates) obtained for each object were compared in a 2 (object size) × 2 (object weight) × 3 (cTBS group) mixed analysis of variance (ANOVA). Here, object size and object weight were within factors and cTBS group was a between factor.

Second, in addition to the effects on the average of all trials, we tested the performance on the first trials only. This was done to test how forces were scaled to object appearance without any previous experience with the objects. Therefore, we took the last lift of the practice trials (medium light), the first lift (small light), and the second lift (large light) and compared these in a 3 (size) × 3 (cTBS group) ANOVA. Here, size and cTBS group were within and between factors, respectively. For the perceptual estimates, we compared the first and second lift in a 2 (size) × 3 (cTBS group ANOVA), because no perceptual judgment was obtained for the practice trials with the medium weight. For three participants, a measurement error was observed in the first or second trial and these participants were excluded from this analysis.

Finally, we examined the effects of previous lifted weight on current lifted weight. We examined these order effects of object weight since it is known that the weight of previously lifted objects can influence force scaling and weight perception (1, 38). Therefore, we ordered the trials in four possible ways: light-light (LL), heavy-light (HL), light-heavy (LH), and heavy-heavy (HH), regardless of object size. For instance, in a heavy-light order, a heavy object was lifted first and the force and perceptual parameters for lifting the light object on the next trial were examined. We conducted a 2 (current weight) × 2 (previous weight) × (cTBS group) ANOVA on the three force parameters and the perceptual estimates. Here, current weight and previous weight were within factors and cTBS group was a between factor.

The mixed ANOVAs as described earlier were performed to investigate an effect of cTBS. In case of a main effect or interaction with cTBS group, the ANOVA was split into three separate repeated-measures ANOVAs to investigate the effects in each cTBS group. If no effect or interaction of cTBS group was found, the groups were pooled into repeated-measures ANOVA to further investigate possible within-interaction effects. In this case, for all effects of within factors, the results from the pooled ANOVA were reported. Further post-hoc tests were performed using *t* tests with a Bonferroni correction. A *P* value of <0.05 was considered significant.

#### Bayesian statistics.

The absence of a significant effect cannot directly be interpreted as a confirmation of the absence of the effect. It has been suggested that especially in cTBS experiments, additional Bayesian statistics could be helpful (39). Since we were especially interested in the effects of object size and the effect of aIPS, we calculated Bayes factor only for the size × cTBS interaction effects. We only did this for the comparison between the aIPS and the Sham condition, where the influence of cTBS over aIPS was compared with the control condition (Sham), specifically for the differences between the two object sizes. We used the methods as described in Ref. 40 and used the free calculator from Ref. 41. This method requires the before be chosen as a specific distribution with defined lower and upper limits. In short, the raw interaction effects were calculated as the difference between the object sizes, averaged over object weight, in the Sham condition and the aIPS condition. Next, Bayes factor was determined for these size effects. The alternative hypothesis was represented as a uniform distribution with as lower limit the effect of the Sham condition and as upper limit the effect of the aIPS condition. These limits follow from the assumption that cTBS over aIPS could maximally eradicate the baseline effect in the Sham condition, or, maximally increase the baseline effect with the effect in the aIPS condition. We chose a uniform distribution, because we had no specific indication for a likely effect size. The null hypothesis assumes no difference between the conditions. A Bayes factor lower than 1/3 would be interpreted as evidence for the null hypothesis, whereas a Bayes factor higher than 3 would be interpreted as evidence for the alternative (42).

#### Correlations.

To test whether the effects on force scaling and perceptual estimates were related, we performed Pearson’s correlations between these parameters. All parameters were converted into *z* scores. Then, we performed correlations between effects of size and effects of sensorimotor memory. For the effects of size, we subtracted trials with big objects from small objects for light and heavy objects separately (i.e., small-light − big-light and small-heavy − big-heavy). We correlated these differences for the perceptual estimates with the differences for GFR1st, LFR1st, and LPD. The correlations were performed for each cTBS group separately. Similarly, for the sensorimotor memory effects, we subtracted the values for trials that had a previous light object with trials with a previous heavy object for current light and heavy objects separately (i.e., HL-LL and HH-LH). We correlated these differences for the perceptual estimates with those for the three force parameters. To adjust for the multiple comparisons, we used a Bonferroni correction for the 18 correlations (3 cTBS groups × 3 variables × 2 weights).

We also performed trial-by-trial correlations for each participant between perceptual estimates and GFR1st, LFR1st, and LPD. We did this for the light and heavy object set separately, to investigate that effects of size were not affected by object weight. To adjust for the multiple comparisons, we used a Bonferroni correction for the 18 correlations.

## RESULTS

In the present study, we investigated the effects of cTBS over aIPS on the size-weight illusion and fingertip force scaling. Participants first received cTBS over aIPS, M1, or Sham stimulation. The experiment then proceeded with participants lifting objects of different sizes and weight and estimating their heaviness. All data used for analyses and figures can be found in Supplemental Data (all Supplemental data and Figures are available at https://doi.org/10.17605/OSF.IO/U9VQ4).

### MEPs

After careful observation of the MEP data, it appeared that the electrodes used for recording MEPs might not have produced reliable results. We therefore report the MEP results in Supplemental Fig. S1 for transparency reasons, but will not further discuss or analyze them.

### Perceptual Estimates Were Not Affected by cTBS

**Figure 2.**
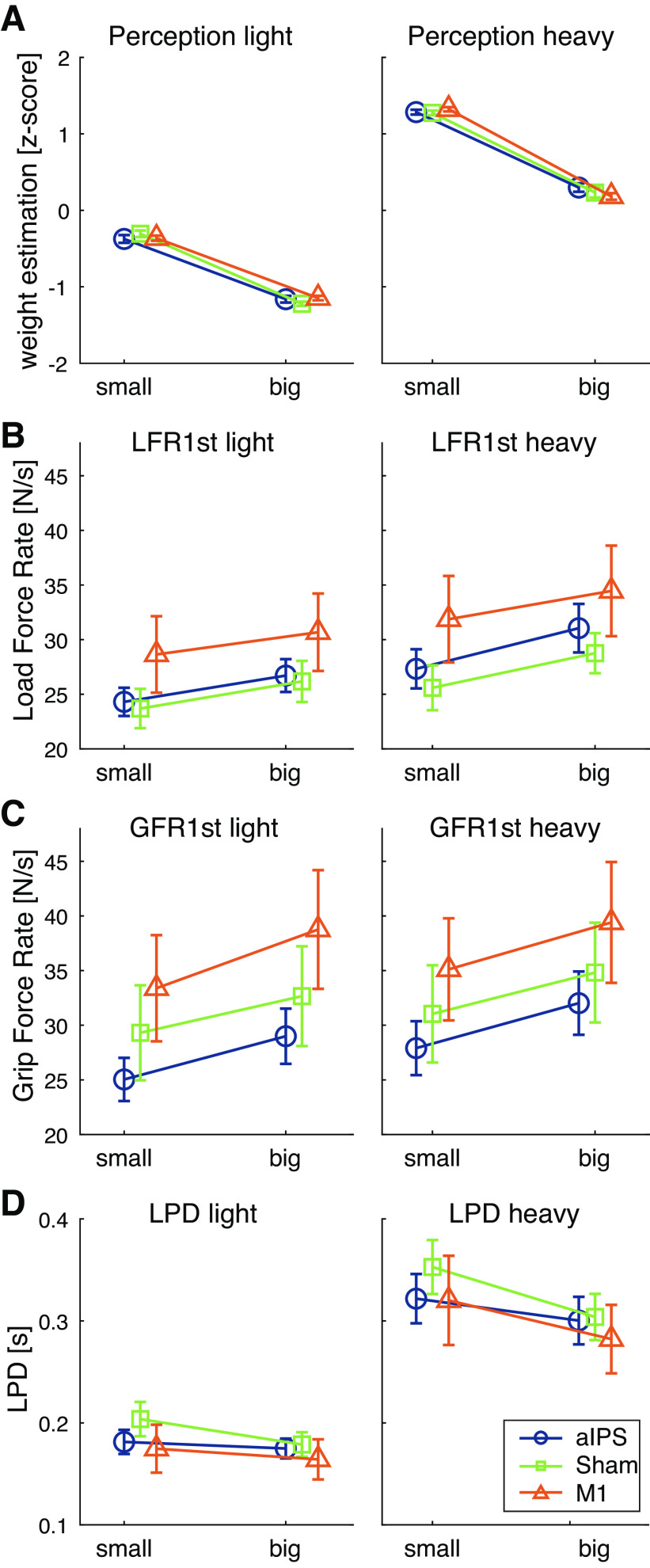
Results for perceptual estimates (*A*), first peak of load force rate (LFR1st; *B*) first peak of grip force rate (GFR1st; *C*), and load force duration (LPD; *D*). Values are shown for light (*left*) and heavy objects (*right*), for small and big objects, and for each continuous theta burst stimulation (cTBS) group separately [perception: anterior intraparietal sulcus (aIPS), *n* = 16; Sham, *n* = 15; M1, *n* = 16. Force parameters: *n* = 15 for all groups]. Error bars represent standard errors of the mean. Main effects of size and mass were found for all parameters (repeated-measures ANOVA).

For the normalized perceptual estimates, the judgments were compared for each object (Fig. 2). The 2 (mass) × 2 (size) × 3 (cTBS group) ANOVA showed the effects of size, mass, and mass × size, but did not show an effect or interaction with cTBS group. Therefore, a 2 (mass) × 2 (size) ANOVA was performed on the pooled groups. This showed an effect of mass [*F*(1,46) = 5,557.7, *P* < 0.001, $\eta_{\text{p}}^{2}$ = 0.99], size [*F*(1,46) = 1161.9, *P* < 0.001, $\eta_{\text{p}}^{2}$ = 0.96], and an interaction of mass × size [*F*(1,46) = 13.3, *P* = 0.001, $\eta_{\text{p}}^{2}$ = 0.23]. Post hoc effects indicated that the light object was perceived to be lighter than the heavy object for both small (*P* < 0.001) and large (*P* < 0.001) sets, as expected in normal weight perception. Furthermore, a size-weight illusion was seen, with small objects perceived as heavier than large objects, both for the light (*P* < 0.001) and heavy (*P* < 0.001) object set. The interaction appears to be explained by a larger-magnitude SWI in heavy objects.

**Figure 3.**
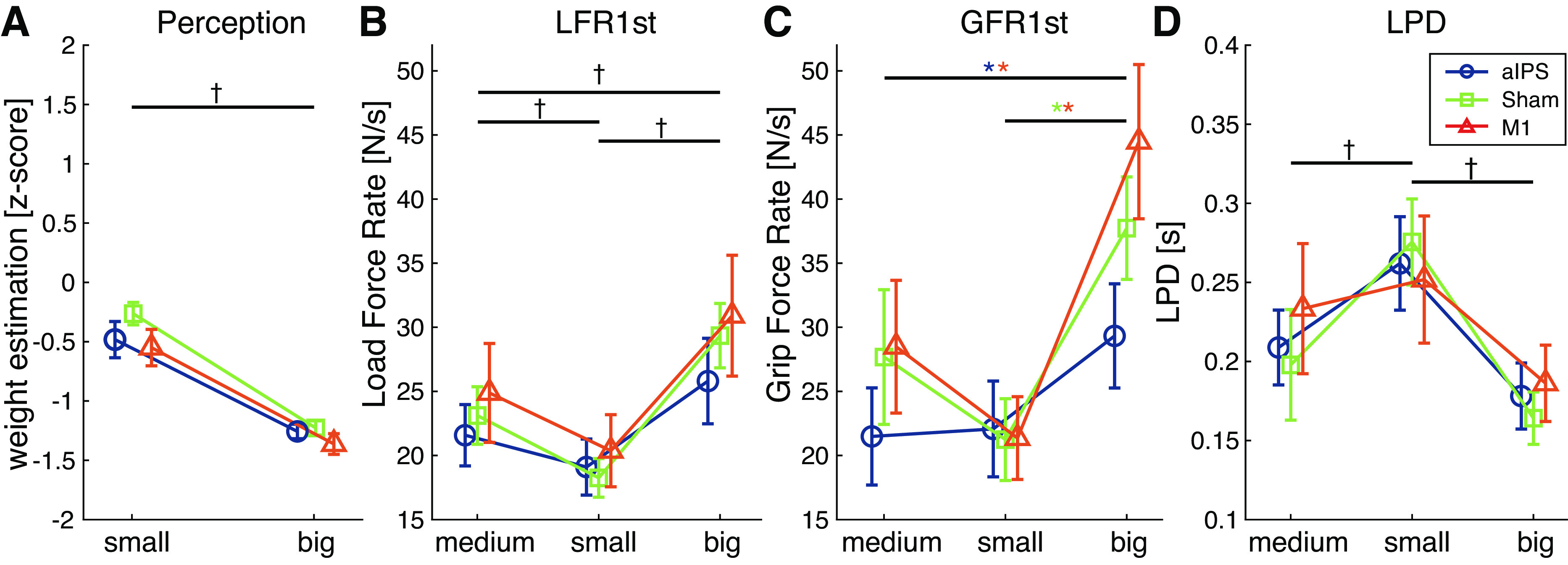
Results for the first two trials (small and big object) for perceptual estimates (*A*), first peak of load force rates (LFR1st; *B*) and grip force rates (GFR1st; *C*), and loading phase durations (LPD; *D*). For the force parameters, also the last practice trial (medium object) is shown. Values are shown for each continuous theta burst stimulation (cTBS) group [perception: anterior intraparietal sulcus (aIPS), *n* = 14; Sham, *n* = 14; M1, *n* = 16. Force parameters: aIPS, *n* = 13; Sham, *n* = 14; M1, *n* = 15]. Error bars represent standard error of the mean. Effects are shown for the repeated measures ANOVA. †Main effect of size. *Colors denote cTBS burst stimulation group-specific effects of object size.

For the first two trials, the two differently sized objects were also perceived differently (Fig. 3). The 2 (size) × 3 (cTBS group) ANOVA revealed an effect of size [*F*(1,41) = 146.6, *P* < 0.001, $\eta_{\text{p}}^{2}$ = 0.78]. The small object was perceived to be heavier than the large object. There was no effect of cTBS group or an interaction.

**Figure 4.**
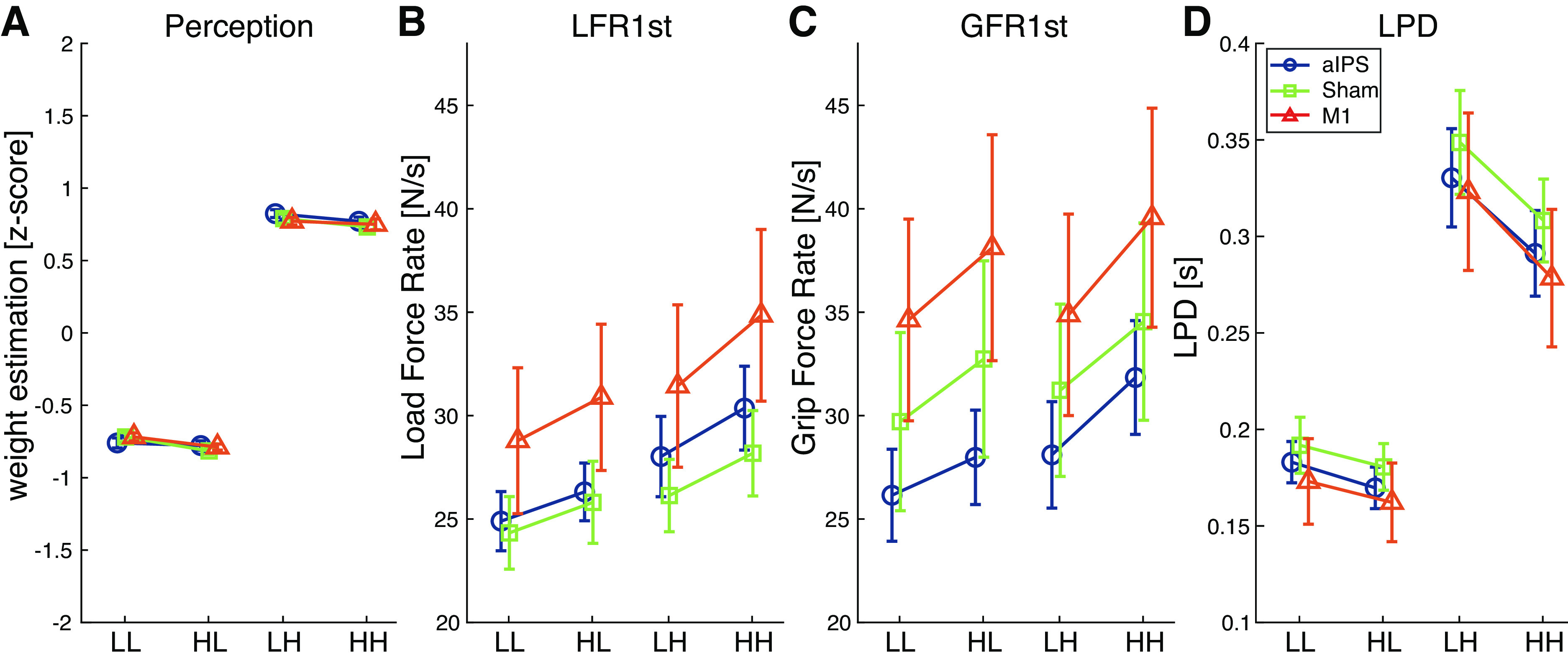
Order effects for perceptual estimates (*A*), first peak of load force rates (LFR1st; *B*) and grip force rates (GFR1st; *C*) and loading phase durations (LPD; *D*). Values are shown for the object orders light-light (LL), heavy-light (HL), light-heavy (LH), and heavy-heavy (HH), for each continuous theta burst stimulation (cTBS) group [perception: anterior intraparietal sulcus (aIPS), *n* = 16; Sham, *n* = 15; M1, *n* = 16. Force parameters: *n* = 15 for all groups]. Error bars represent standard error of the mean. Effects of previous and current weight were found for all parameters (repeated-measures ANOVA).

Finally, we analyzed order effects in the perceptual estimates, represented in Fig. 4. The 2 (current weight) × 2 (previous weight) × 3 (cTBS group) ANOVA had no effects or interactions with cTBS group, but did show effects of current and previous weight. A 2 × 2 ANOVA pooled over the cTBS groups showed main effects of current weight [*F*(1,46) = 5306.5, *P* < 0.001, $\eta_{\text{p}}^{2}$ = 0.99] and previous weight [*F*(1,46) = 9.6, *P* = 0.003, $\eta_{\text{p}}^{2}$ = 0.17], without an interaction. Again, light objects were perceived as lighter than heavy objects. Furthermore, when a heavy object was previously lifted, objects felt lighter than when a light object was previously lifted. These findings indicate that participants experienced a size-weight illusion, already in the first trials, and replicate the perceptual bias found in Ref. 38. However, these perceptual estimates were not affected by cTBS.

### Force Parameters

#### Objects were not lifted differently in response to cTBS.

Results for the force parameters are shown in Fig. 2. In the analysis for all objects, no main effects of cTBS group were found for LFR1st, GFR1st, or LPD, but effects of mass and size were found. Therefore, 2 (mass) × 2 (size) ANOVAs were performed on the pooled data. For LFR1st, main effects of mass [*F*(1,44) = 58.2, *P* < 0.001, $\eta_{\text{p}}^{2}$ = 0.57] and size [*F*(1,44) = 54.2, *P* < 0.001, $\eta_{\text{p}}^{2}$ = 0.55] were found, where force rates were higher for heavy and large objects compared with light and small objects, respectively.

For GFR1st, the ANOVA revealed an effect of mass [*F*(1,44) = 43.3, *P* < 0.001, $\eta_{\text{p}}^{2}$ = 0.50], where GFR1st was higher for heavy objects compared with light objects. Furthermore, an effect of size [*F*(1,44) = 51.3, *P* < 0.001, $\eta_{\text{p}}^{2}$ = 0.54] showed that GFR1st was higher for larger than smaller objects.

For LPD, main effects of mass [*F*(1,44) = 223.6, *P* < 0.001, $\eta_{\text{p}}^{2}$ = 0.84], size [*F*(1,44) = 26.8, *P* < 0.001, $\eta_{\text{p}}^{2}$ = 0.38], and an interaction of mass × size [*F*(1,44) = 14.4, *P* < 0.001, $\eta_{\text{p}}^{2}$ = 0.25] were found. Post hoc tests indicated that the light objects were lifted with shorter LPDs than heavy objects, both for small (*P* < 0.001) and large (*P* < 0.001) objects. In addition, the smaller objects were lifted with longer LPDs than the large objects, both in the light (*P* = 0.020) and heavy (*P* < 0.001) object set. The interaction did not reveal new insights, but might be explained by larger effects of size for heavy objects.

In sum, it seemed that force scaling was adapted to object size and mass, but this was not affected by cTBS.

#### cTBS influenced grip force scaling when lifting without object experience.

The results for the last practice trial and the first two trials are shown in Fig. 3. Individual values for small and big objects are shown in Supplemental Fig. S2. For LFR1st, the first trials were only affected by size [*F*(2,78) = 26.4, *P* < 0.001, $\eta_{\text{p}}^{2}$ = 0.40], with no main effect or interaction with cTBS group. Post hoc analyses showed that all sizes differed, where large objects were being lifted with higher force rates compared with small (*P* < 0.001) and medium sizes (*P* = 0.001), and the medium object was lifted with a higher LFR1st than the small one (*P* = 0.004).

For GFR1st in the first trials, the 3 (size) × 3 (cTBS group) demonstrated a main effect of size [*F*(2,78) = 39.4, *P* < 0.001, $\eta_{\text{p}}^{2}$ = 0.50], but also an interaction of size × cTBS group [*F*(4,78) = 3.3, *P* = 0.014, $\eta_{\text{p}}^{2}$ = 0.15]. Therefore, we performed separate ANOVAs for each cTBS group. We found effects of size in each cTBS group [aIPS: *F*(2,24) = 6.3, *P* = 0.007, $\eta_{\text{p}}^{2}$ = 0.34; M1: *F*(2,28) = 28.4, *P* < 0.001, $\eta_{\text{p}}^{2}$ = 0.67; Sham: *F*(2,26) = 10.6, *P* < 0.001, $\eta_{\text{p}}^{2}$ = 0.45]. However, post hoc analysis showed different results. In the aIPS group, only a difference between the medium and large object was found (*P* = 0.027), where GFR1st was greater for the large object. No significant differences were found between small and medium (*P* = 1.00) or small and big (*P* = 0.053) objects. In the M1 group, the large object had a higher GFR1st compared with both the small (*P* < 0.001) and medium (*P* < 0.001) object. Small and medium objects were not significantly different (*P* = 0.062). Finally, with Sham stimulation, the GFR1st differed only between the small and large object (*P* = 0.001), with a larger GFR1st for the large object. Differences between small and medium (*P* = 0.161) or medium and large objects (*P* = 0.12) were not significant. No differences between the cTBS groups were found for any of the object sizes.

For LPD, only a main effect of size [*F*(2,78) = 15.2, *P* < 0.001, $\eta_{\text{p}}^{2}$ = 0.28] was found, where small objects had shorter LPDs than medium (*P* = 0.012) and large (*P* < 0.001) objects.

To summarize, on the first trials forces were already scaled toward object size. When cTBS was applied, this affected the grip force scaling toward object size differently in the aIPS, M1, and Sham group. Specifically, no difference in grip force rate between small and large objects was found after cTBS on aIPS.

#### Force scaling according to previous object weight was largely unaffected by cTBS.

Effects of object weight order are shown in Fig. 4. The analysis on object weight order on the GFR1st revealed main effects of current weight [*F*(1,42) = 36.5, *P* < 0.001, $\eta_{\text{p}}^{2}$ = 0.47], previous weight [*F*(1,42) = 76.6, *P* < 0.001, $\eta_{\text{p}}^{2}$ = 0.65], but also interactions of previous weight × current weight [*F*(1,42) = 4.1, *P* = 0.049, $\eta_{\text{p}}^{2}$ = 0.09] and current weight × cTBS group [*F*(2,42) = 4.0, *P* = 0.025, $\eta_{\text{p}}^{2}$ = 0.16]. To analyze the interaction with cTBS group, we performed separate 2 × 2 ANOVAs for each cTBS group. For all cTBS groups, a main effect of current weight was found [aIPS: *F*(1,14) = 21.4, *P* < 0.001, $\eta_{\text{p}}^{2}$ = 0.61; M1: *F*(1,14) = 5.1, *P* = 0.041, $\eta_{\text{p}}^{2}$ = 0.27; Sham: *F*(1,14) = 10.3, *P* = 0.006, $\eta_{\text{p}}^{2}$ = 0.42], where the light objects had a lower GFR1st than heavy objects. In addition, for all cTBS groups, a main effect of previous weight was found [aIPS: *F*(1,14) = 34.9, *P* < 0.001, $\eta_{\text{p}}^{2}$ = 0.71; M1: *F*(1,14) = 34.3, *P* < 0.001, $\eta_{\text{p}}^{2}$ = 0.71; Sham: *F*(1,14) = 16.5, *P* = 0.001, $\eta_{\text{p}}^{2}$ = 0.54]. Here, a previous light weight resulted in a lower GFR1st than a previous heavy weight. No interaction between current and previous weight was found in any cTBS group. Also, no differences between the cTBS groups were found for light nor heavy current weights. Therefore, this interaction between current weight and cTBS group is difficult to explain. From Fig. 4, it appears that the difference between light and heavy objects is slightly reduced after cTBS on M1 and increased after cTBS on aIPS.

For the LFR1st and LPD, no main or interaction effects with cTBS group were found, but we did see effects of current and previous weight and interactions between those within factors. Therefore, the data were pooled over cTBS group in a 2 × 2 ANOVA. For LFR1st, main effects of current weight [*F*(1,44) = 53.2, *P* < 0.001, $\eta_{\text{p}}^{2}$ = 0.55], previous weight [*F*(1,44) = 46.6, *P* < 0.001, $\eta_{\text{p}}^{2}$ = 0.51], and an interaction of current weight × previous weight were found [*F*(1,44) = 4.9, *P* = 0.031, $\eta_{\text{p}}^{2}$ = 0.10]. Post hoc analysis indicated that LFR1st was higher for current heavy than current light objects, both when previously heavy (*P* < 0.001) or light objects (*P* < 0.001) were lifted. Furthermore, if previously a heavy object was lifted, LFR1st was higher compared with previously lifting a light object, both for current light (*P* < 0.001) and heavy objects (*P* < 0.001). For LPD, main effects of current weight [*F*(1,44) = 234.2, *P* < 0.001, $\eta_{\text{p}}^{2}$ = 0.84), previous weight [*F*(1,44) = 80.7, *P* < 0.001, $\eta_{\text{p}}^{2}$ = 0.65], and an interaction of current weight × previous weight [*F*(1,44) = 28.1, *P* < 0.001, $\eta_{\text{p}}^{2}$ = 0.39] were found. Post hoc analyses revealed that the LPD was shorter for lifting light objects compared with heavy ones, both when the previous object was light (*P* < 0.001) or heavy (*P* < 0.001). When the previous object was light, LPDs were longer, both when the current object was light (*P* < 0.001) and heavy (*P* < 0.001). The interactions did not reveal new insights, but could result from slightly larger order effects for heavy objects than light ones.

In sum, force scaling did also depend on the weight of the previous lifted object. However, this effect was not influenced by cTBS.

### Correlations between Force Parameters and Perceptual Estimates

**Figure 5.**
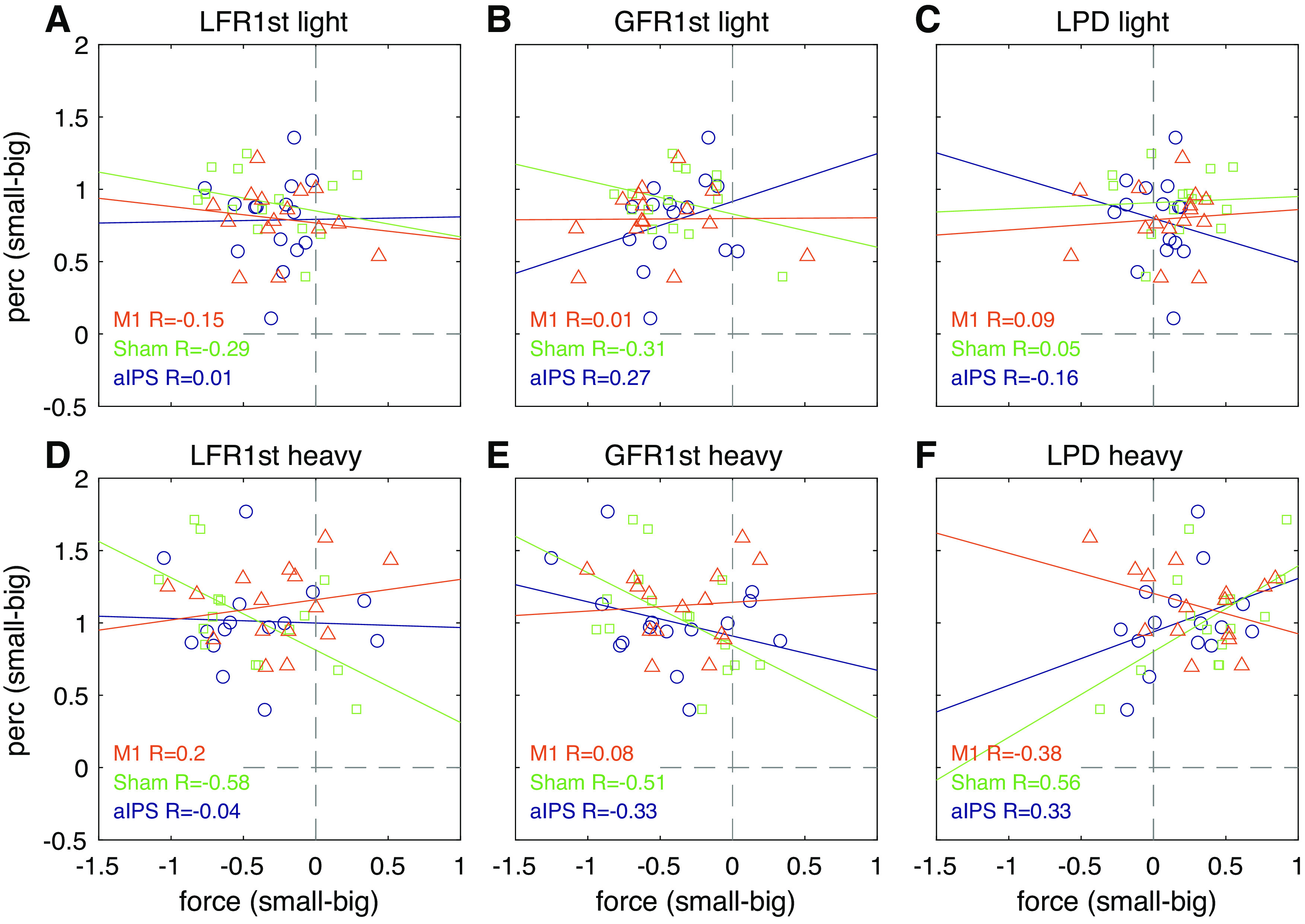
Pearson’s correlations between size effects of perception and size effects on force parameters first peak of load force rate (LFR1st; *A* and *D*) and grip force rate (GFR1st; *B* and *E*) and loading phase duration (LPD; *C* and *F*). Values are shown for light (*A*–*C*) and heavy (*D*–*F*), for each continuous theta burst stimulation (cTBS) group separately (all *n* = 15). Dashed gray lines indicate the point of no size difference. Values above or below the horizontal line indicate higher or lower estimates for small objects, respectively. Values to the left or right of the horizontal line indicate lower or higher force rates, or shorter or longer LPDs for small objects, respectively. aIPS, anterior intraparietal sulcus.

To test whether the effects of object size on the force scaling and perceptual ratings were related, we calculated correlations of the size effects on force parameters and perceptual estimates (Fig. 5). No significant correlations were observed (all *P* > 0.42). Considering the strict Bonferroni correction (dividing *P* value by 18 to obtain a threshold of 0.0028), we also looked at the uncorrected values. Without a correction, two significant correlations were found in the Sham group when lifting heavy objects: a negative relation was found between LFR1st and perception (*R* = −0.58, *P* = 0.023) and between LPD and perception (*R* = 0.56, *P* = 0.029). These relations indicate that the amount of scaling to size in force parameters is related to the strength of the SWI. However, given only 2 out of 18 correlations were significant when uncorrected, this does not provide strong evidence for a relation between the effects of size on force scaling and perceptual ratings.

Average trial-by-trial correlations are shown in Table 1, where many *R* values were significantly different from zero for the force rate parameters. Since the correlations were performed separately between object weights, variations in values will most likely reflect variations due to object size. Hence, a significant correlation suggests a relation between force scaling to size and SWI strength. However, no apparent differences were visible between the cTBS groups.

**Table 1.** Mean R values for trial-by-trial Pearson’s correlations between perceptual estimates and force scaling parameters for light and heavy objects separately

	Light			Heavy		
	LFR1st	GFR1st	LPD	LFR1st	GFR1st	LPD
aIPS	−0.20 ± 0.04*	−0.20 ± 0.03*	0.09 ± 0.04	−0.19 ± 0.04*	−0.24 ± 0.04*	0.10 ± 0.04
Sham	−0.22 ± 0.05*	−0.20 ± 0.04*	0.11 ± 0.06	−0.21 ± 0.06	−0.19 ± 0.05	0.25 ± 0.06*
M1	−0.20 ± 0.05*	−0.29 ± 0.05*	0.13 ± 0.08	−0.18 ± 0.05	−0.22 ± 0.06*	0.19 ± 0.06

Values indicate means ± standard error. aIPS, anterior intraparietal sulcus; GFR1st, first peak of grip force rate; LFR1st, first peak of load force rate; LPD, and load force duration.

* *P* < 0.05 significant from zero with one-sample *t* test, corrected for multiple comparisons.

Furthermore, to test whether the order of object weight similarly affected force parameters and perceptual estimations, we examined whether the sensorimotor memory effects correlated with order effects on the perceptual estimates; none of these correlations were significant (all *P* = 1.00). Even when no Bonferroni correction was applied, no significant effects were found.

### Bayesian Statistics on Size × cTBS Interaction

To determine the Bayes factor for the size × cTBS interaction, we first determined the *F* value of this effect for aIPS and Sham only. We performed a 2 (mass) × 2 (size) × 2 (cTBS) mixed ANOVA on the perceptual *z* scores, GFR1st, LFR1st, and LPD. We found the following results for the size × cTBS interaction: perception [*F*(1,29) = 1.1, *P* = 0.302, $\eta_{\text{p}}^{2}$ = 0.04], GFR1st [*F*(1, 28) = 0.2, *P* = 0.691, $\eta_{\text{p}}^{2}$ = 0.01], LFR1st [*F*(1, 28) = 0.1, *P* = 0.741, $\eta_{\text{p}}^{2}$ = 0.00], and LPD [*F*(1, 28) = 5.0, *P* = 0.034, $\eta_{\text{p}}^{2}$ = 0.15]. Note that the effect was significant for LPD, but not for the other variables. This resulted in Bayes factors of 0.17 for perception, 0.32 for LFR1st, 0.42 for GFR1st, and 5.55 for LPD. These values indicate evidence for the null-hypothesis for perceptual estimates and LFR1st, but inconclusive evidence for GFR1st. By contrast, for LPD, there is substantial evidence for the alternative hypothesis, indicating an effect of cTBS over aIPS on anticipatory force scaling to size.

Furthermore, we also performed this analysis on the data of the first two trials. This represents the effect of object size (small or heavy) and cTBS (aIPS vs. Sham). A new 2 (size) × 2 (cTBS) mixed ANOVA on the first two trials gave the following results: perception [*F*(1,26) = 1.1, *P* = 0.295, $\eta_{\text{p}}^{2}$ = 0.04], GFR1st [*F*(1,25) = 4.8, *P* = 0.038, $\eta_{\text{p}}^{2}$ = 0.16], LFR1st [*F*(1,25) = 2.6, *P* = 0.119, $\eta_{\text{p}}^{2}$ = 0.09], and LPD [*F*(1,25) = 0.6, *P* = 0.428, $\eta_{\text{p}}^{2}$ = 0.03]. Note that the effect was only significant in GFR1st, also found in the original analysis. The resulting Bayes factors were 0.45 for perception, 1.40 for LFR1st, 4.71 for GFR1st, and 0.60 for LPD. For GFR1st this gave substantial evidence for the alternative hypothesis, confirming the significant *P* value. For the other variables, inconclusive evidence was found.

## DISCUSSION

The aim of the present study was to evaluate the role of the anterior intraparietal sulcus (aIPS) in anticipatory force scaling to object size and the size-weight illusion (SWI). We used noninvasive brain stimulation to disrupt aIPS before a behavioral task. More specifically, continuous theta burst stimulation (cTBS) was applied over aIPS and compared with two control conditions, where cTBS was either applied over the primary motor cortex (M1) or a Sham stimulation was performed. In the behavioral task, participants lifted objects of different sizes and weights while their fingertip forces were measured and after each lift, they reported felt object heaviness. We found no effect of aIPS stimulation on the SWI and only effects on anticipatory force scaling to size in the first trials. This suggest that aIPS is unlikely to be causally involved in the SWI, and appears to play a transient, but not primary, role in anticipatory force scaling based on visual cues to object size.

A robust SWI was observed in our experiments, with smaller objects perceived to be heavier than large objects of the same mass, in both the light and heavy object set. The SWI effect was not affected by cTBS applied over aIPS nor M1. To further affirm these nonsignificant results, we calculated Bayes factors and these indicated support for the null hypothesis. Therefore, we conclude that aIPS does not play a role in the SWI. Previous clinical evidence suggested a possible role for the posterior parietal cortex, but results were mixed (25, 26). It seems likely instead that other areas are more involved in the SWI, such as the ventral premotor cortex (19) or the lateral occipital cortex (43), although lesions in this latter area did not seem to affect the SWI (44).

When lifting objects of different sizes, participants scaled their forces to object size, with higher rates of force used to lift large objects compared with small objects. We found these effects for the first trials, but surprisingly they were maintained over the course of the experiment. Previous research showed that forces adapted to actual object weight after some experience with the objects (10, 11, 45). However, in these earlier studies objects all had the same weight and were presented in alternating order. An earlier study indicated that a random presentation can induce force scaling to object size compared with lifting object in a consecutive order (46). In the present experiment, two sets of object weight were presented in a random order. Participants could not use earlier experience with the objects to accurately predict object weight, since an object of a specific size could be either light or heavy and, therefore, they might still have relied on size priors to scale their forces. Even though participants could not correctly anticipate the weight because of this random presentation of objects and would often need to correct their forces, they still planned their fingertip forces both based on the size of the current object and on the sensorimotor memory of the previous object.

Contrary to our hypothesis, we did not find large effects of aIPS cTBS on anticipatory force scaling to size. Grip force scaling was reduced in the first trials and the loading phase duration appeared to be slightly altered, but overall effects were minor over the course of the experiment. Although aIPS plays a role in force scaling (17, 36), it might only be transiently involved in anticipatory force scaling to object size, which is mostly visible in the first trials. When interacting with a new object, there is no prior sensorimotor experience and only visual properties, such as size, can be used for anticipatory force scaling. This initial sensorimotor mapping might be controlled by aIPS. After further experience with the objects, other brain areas might be involved in storing this newly learned sensorimotor mapping and integrating this information for generating forces according to available visual cues. Considering the large network of areas involved in the planning and control of grasping behavior (47), anticipatory scaling could be governed by other areas than aIPS, such as the premotor cortex (17, 31, 32). Moreover, it is likely that aIPS is more concerned with online control and error corrections, such as known for grasp parameters (21, 48–50) and less with anticipatory force scaling to object properties.

The transient effect of aIPS on anticipatory force scaling in the first trials was only found for the grip force rate, but not for the load force rate. Although these two measures are often coupled (1, 51, 52), it has also been shown that this coupling can be intermittent (53). Furthermore, previous TMS studies found a role for different brain areas specifically in grip forces, but not in load forces (32, 54). Of particular interest, two studies found an effect of TMS over aIPS only in grip force, but not in load force scaling (36, 55). A possible explanation for this discrepancy might be attributed to the different roles of the two force components. Although the load force is more tightly coupled to object weight, the grip force is also adjusted to the frictional properties of the objects and includes a safety margin with respect to the object weight to avoid slipping. Therefore, this force component can be more flexibly adjusted than the load force. Furthermore, because of this flexibility, the grip force might also be more easily susceptible to external influences, such as the disruptions caused by TMS.

In the present study, the size of the object was visually shown to the participants, but they had no access to haptic information about the size since they lifted the object with two force sensors. Previous research has shown that the size-weight illusion is stronger when size information is presented haptically, or in combination with vision (6, 7). Possibly, a stronger effect of cTBS over aIPS would have been found if the size was also presented haptically, since this area is also known to be multimodal (56). However, given the strong evidence we found that there was no effect of cTBS over aIPS on the size-weight illusion, it remains doubtful whether an effect would be found when haptic information was provided. Since we only found a transient effect of aIPS on anticipatory force scaling, the addition of haptic information, which would only be available after experience with the objects, might not have contributed to the effect on anticipatory force scaling.

Several previous studies showed that fingertip forces and perceptual estimates adapt differently to repeated object lifting with SWI objects (10–12, 57, 58), where the illusion is still present after several lifts but the forces are scaled correctly for the equally weighting objects. Interestingly, we did not only find that the anticipatory scaling to object size remained when objects were presented randomly, but we also found correlations with perceptual effects. However, these effects should be interpreted with caution, since we only observed small correlations in trial-by-trial comparisons and no significant between-subject correlations. Furthermore, there appeared to be no difference between cTBS conditions when correlating individual trials. Although this relation requires further research, it is noteworthy that Gordon et al. (46) observed that participants who did not show an SWI, also showed a probing strategy with little force scaling to size, further suggesting links between lifting behavior and perceptual estimates.

Finally, we did not find the effects of M1 stimulation. Stimulation of M1 did not affect sensorimotor memory, whereas this was shown in previous studies (31, 59). However, it has been acknowledged that cTBS effects can be very variable and are not found in each participant (60, 61). In addition, since objects also varied in size, it is possible that sensorimotor memory effects were weaker, as forces were also scaled to object size and not only to previous experienced weight. With more variability in force parameters, small effects of M1 stimulation on sensorimotor memory might have been masked.

The lack of effects of M1 stimulation on force scaling could suggest that our cTBS paradigm was not effective. However, we argue that this is unlikely to be the case. We used standard protocols similar to studies that previously showed differences between TMS and Sham stimulation with a reduced intensity (32, 33). Recently, it was shown in nonhuman primates that high-intensity TMS induced neural spikes, whereas low-intensity TMS did not (34, 35). Therefore, we assume that our cTBS protocol was effective, but did not always induce visible effects on object lifting behavior. Finally, although we did not find effects of M1 stimulation, we did find differences in force scaling after aIPS stimulation, indicating that our protocol was indeed effective, but only transient behavioral effects were observed.

To conclude, we investigated the role of aIPS in force scaling to size and the SWI. Although aIPS might play a transient role in the initial scaling grip forces to object size, it does not seem to be involved in mediating the SWI.

## SUPPLEMENTAL DATA

10.17605/OSF.IO/U9VQ4 Supplemental data and Supplemental Figs. S1 and S2: https://doi.org/10.17605/OSF.IO/U9VQ4.

## GRANTS

This research was supported by Fonds Wetenschappelijk Onderzoek Grants FWO post-doctoral fellowship, Belgium, 12X7118N (to V. van Polanen) and FWO Odysseus, Belgium, G/0C51/13N (to M. Davare).

## DISCLOSURES

No conflicts of interest, financial or otherwise, are declared by the authors.

## AUTHOR CONTRIBUTIONS

V.v.P., G.B., and M.D. conceived and designed research; V.v.P. performed experiments; V.v.P. analyzed data; V.v.P., G.B., and M.D. interpreted results of experiments; V.v.P. prepared figures; V.v.P. drafted manuscript; V.v.P., G.B., and M.D. edited and revised manuscript; V.v.P., G.B., and M.D. approved final version of manuscript.

## ENDNOTE

At the request of the authors, readers are herein alerted to the fact that additional materials related to this manuscript may be found at https://doi.org/10.17605/OSF.IO/U9VQ4. These materials are not a part of this manuscript and have not undergone peer review by the American Physiological Society (APS). APS and the journal editors take no responsibility for these materials, for the website address, or for any links to or from it.
